# Targeting ASCT2‐mediated glutamine uptake blocks prostate cancer growth and tumour development

**DOI:** 10.1002/path.4518

**Published:** 2015-04-07

**Authors:** Qian Wang, Rae‐Anne Hardie, Andrew J Hoy, Michelle van Geldermalsen, Dadi Gao, Ladan Fazli, Martin C Sadowski, Seher Balaban, Mark Schreuder, Rajini Nagarajah, Justin J‐L Wong, Cynthia Metierre, Natalia Pinello, Nicholas J Otte, Melanie L Lehman, Martin Gleave, Colleen C Nelson, Charles G Bailey, William Ritchie, John EJ Rasko, Jeff Holst

**Affiliations:** ^1^ Origins of Cancer LaboratoryCentenary InstituteCamperdownNSWAustralia; ^2^ Sydney Medical SchoolUniversity of SydneyNSWAustralia; ^3^ Gene and Stem Cell Therapy ProgramCentenary InstituteCamperdownNSWAustralia; ^4^ Discipline of Physiology, Bosch Institute and Charles Perkins CentreUniversity of SydneyNSWAustralia; ^5^ BioinformaticsCentenary InstituteCamperdownNSWAustralia; ^6^ Department of Urologic SciencesUniversity of British ColumbiaVancouverBCCanada; ^7^ Australian Prostate Cancer Research Centre–QueenslandQueensland University of TechnologyAustralia; ^8^ Cell and Molecular TherapiesRoyal Prince Alfred HospitalCamperdownNSWAustralia

**Keywords:** ASCT2, SLC1A5, glutamine, cell cycle, metabolism, prostate cancer

## Abstract

Glutamine is conditionally essential in cancer cells, being utilized as a carbon and nitrogen source for macromolecule production, as well as for anaplerotic reactions fuelling the tricarboxylic acid (TCA) cycle. In this study, we demonstrated that the glutamine transporter ASCT2 (SLC1A5) is highly expressed in prostate cancer patient samples. Using LNCaP and PC‐3 prostate cancer cell lines, we showed that chemical or shRNA‐mediated inhibition of ASCT2 function in vitro decreases glutamine uptake, cell cycle progression through E2F transcription factors, mTORC1 pathway activation and cell growth. Chemical inhibition also reduces basal oxygen consumption and fatty acid synthesis, showing that downstream metabolic function is reliant on ASCT2‐mediated glutamine uptake. Furthermore, shRNA knockdown of ASCT2 in PC‐3 cell xenografts significantly inhibits tumour growth and metastasis in vivo, associated with the down‐regulation of E2F cell cycle pathway proteins. In conclusion, ASCT2‐mediated glutamine uptake is essential for multiple pathways regulating the cell cycle and cell growth, and is therefore a putative therapeutic target in prostate cancer. © 2015 The Authors. The Journal of Pathology published by John Wiley & Sons Ltd on behalf of Pathological Society of Great Britain and Ireland.

## Introduction

Glutamine is the most abundant amino acid in plasma, functioning as a critical source of nitrogen and carbon in cells. The metabolic shift in cancer, however, imparts a new ability to the cells to utilize glutamine as an alternative fuel source to glucose for the tricarboxylic acid (TCA) cycle, and as a source of fatty acid production through reductive carboxylation 1, 2, 3. Many of these changes in glutamine metabolism result directly from oncogenic transformation, such as Myc amplification 4, 5, 6, commonly seen in prostate cancer 7.

Another important role for amino acids is to control signalling through the nutrient sensor mTORC1. The tumour suppressor gene, *PTEN*, is commonly mutated or deleted in prostate cancer 8, leading to up‐regulation of the PI3K–Akt–mTORC1 signalling pathway. The amount of intracellular amino acids, such as leucine, determines the activity of mTORC1 through Rag complexes on the lysosome surface 9, 10, 11, 12, 13. In combination with leucine, glutamine has also been shown to be critical for mTORC1 signalling by enhancing glutaminolysis and α‐ketoglutarate production 14. This link is further supported by the role of glutamine in facilitating leucine transport 15 and the feedback generated by activation of mTORC1, which promotes glutaminolysis in cancer 3.

The major glutamine transporter in cancer cells is alanine–serine–cysteine transporter‐2 (ASCT2; SLC1A5) 26, 27, 28. ASCT2 is a Na^+^‐dependent, broad‐scope neutral amino acid exchanger that belongs to solute carrier (SLC) family‐1, which mediates the obligatory exchange of substrate amino acids, including alanine, serine, cysteine, threonine, glutamine and asparagine 16. ASCT2 is expressed in the normal prostate and in prostate cancer 17. Previous studies have shown that ASCT2 function is important for cancer cell growth in melanoma 18, acute myeloid leukaemia 19, lung cancer 20, neuroblastoma 21 and pancreatic ductal carcinoma 22.

In this study we showed that ASCT2 expression is increased in prostate cancer patient samples, and that knockdown inhibits cell cycle progression, prostate cancer growth and spontaneous metastasis *in vivo*. ASCT2 chemical inhibitors also suppressed glutamine transport, cell growth and glutamine metabolism *in vitro*. Our study suggests that compounds targeting ASCT2 may provide novel therapeutics for prostate cancer.

## Materials and methods

For additional materials and methods, please see the online Supplementary material.

### Patient specimens

Prostate cancer specimens from radical prostatectomy (*n =* 194) were obtained from the Vancouver Prostate Centre Tissue Bank (http://www.prostatecentre.com/our‐research/core‐facilities/biorepository). This project was approved by the institutional review boards at the University of British Columbia (Vancouver, Canada) and the CHUQ Research Centre (Québec, Canada). Written informed consent was obtained from all participants. The haematoxylin and eosin (H&E)‐stained slides were reviewed and desired areas were identified on paraffin blocks. Tissue microarrays (TMAs) were manually constructed (Beecher Instruments, MD, USA) by punching duplicate cores of 1 mm for each sample, with quantitative analysis calculated from individual cores (individual Gleason scores) or the average of duplicate cores (NHT TMA analysis).

### TMA immunohistochemistry

TMA staining was conducted using a Ventana auto‐stainer, model Discover XT™, with an enzyme‐labelled biotin–streptavidin system and solvent‐resistant DAB Map kit, using a 1:4000 dilution of rabbit anti‐SLC1A5 (HPA035240). Staining was graded by a pathologist (LF), using a semi‐quantitative, four‐point scale, in which 0 represents no staining, 1 faint or focal staining, 2 moderate intensity in at least a quarter of neoplastic cells, and 3 intense staining in the majority of neoplastic cells. Examples of the staining are shown in Figure S1B (see supplementary material).

### Cell culture and uptake assay

Human prostate cancer cell lines LNCaP‐FGC, PC‐3 and DU145 were purchased from ATCC (Rockville, MD, USA). LNCaP cells had been passaged directly from original low‐passage stocks (2009), and we confirmed PC‐3, LNCaP and DU145 cell identity by STR profiling in 2010 and 2014 (Cellbank, Sydney). Cells were cultured in RPMI 1640 medium containing 10% v/v fetal bovine serum (FBS), penicillin–streptomycin solution and 1 mm sodium pyruvate. Cells were maintained at 37 °C in an atmosphere containing 5% CO_2_. Chemicals were diluted as follows, with control wells treated with the appropriate vehicle controls: H‐Ser(Bzl)‐OH (BenSer; Bachem; diluted in H_2_O); l‐γ‐glutamyl‐*p*‐nitroanilide (GPNA; MP Biochemicals; diluted in H_2_O); bicalutamide (Astra Zeneca; diluted in DMSO). The [^3^H]‐l‐leucine and [^3^H]‐l‐glutamine uptake assays were performed as detailed previously 18, 32.

### Knockdown of ASCT2

ASCT2 shRNA knockdown was performed as previously described 18. Two different shRNAs for ASCT2 were used in this study (Sigma): shASCT2#1 (CCGGGCTGCTTATCCGCTTCTTCAACTCGAGTTGAAGAAGCGGATAAGCAGCTTTTTG) and shASCT2#2 (CCGGCTGGATTATGAGGAATGGATACTCGA GTATCCATTCCTCATAATCCAGTTTTTG).

### Gene expression analysis

PC‐3 cells were incubated in the presence or absence of GPNA or BenSer for 48 h, the cells were harvested and total RNA was extracted from them using Trizol. RNA quality was confirmed (RIN values 9.8–10), using RNA 6000 Nano Chips on an Agilent 2100 Bioanalyser (Agilent Technologies). Libraries were prepared using TruSeq Stranded Total RNA kit (Illumina, San Diego, CA, USA) and paired‐end sequencing was performed on an Illumina HiSeq 2500 at the Kinghorn Cancer Centre (Sydney). Paired‐end RNA‐sequencing reads were trimmed and mapped to annotated human genome (hg19/GRCh37.p13), using Tophat2 with default settings. For each gene, the number of reads were counted and normalized to the library size. The threshold for expression was set at five reads in at least one experimental group. Exact tests were applied to compare differences in the means of read‐counts from four replicates between the treated and untreated groups. The gene expression levels were estimated counts/million reads (CPM). The National Center for Biotechnology Information Gene Expression Omnibus number for mRNA sequencing datasets described in this study is GSE65112.

### PC‐3–luc xenografts and bioluminescence imaging

Athymic (*nu/nu*) male nude mice (Animal Resource Centre, Perth, Australia), aged 6–8 weeks, were housed in a specific pathogen‐free facility, in accordance with the University of Sydney animal ethics committee guidelines. Mice were anaesthetized via 2% isoflurane inhalation and received subcutaneous (s.c.) injections of 1 × 10^6^ PC‐3–luc cells resuspended in 100 ml Hanks' balanced salt solution (HBSS). Xenografts were transplanted in both the right and left ventral flanks of mice, as detailed previously 30. Tumour growth was monitored via bioluminescence imaging performed 48 h following cell implantation and biweekly thereafter for 32 days. During the experiments, two shASCT2 mice were dead 4 days after injection, due to fighting; they were not included in the analyses. Anaesthetized mice received intraperitoneal (i.p.) injections of d‐luciferin substrate (150 mg/kg in DPBS; Gold Biotechnology) and images were acquired after a 15 min interval, using the Xenogen *in vivo* imaging system (IVIS) Lumina II (Caliper Life Science, MA, USA). Regions of interest were determined using Living Image software (Caliper Life Science) and quantified in photons/s (p/s). After 32 days, the animals were sacrificed following the final imaging time point. Livers and lungs were removed for IVIS‐Lumina II analysis to detect spontaneous metastases. After being imaged and weighed, tumours were collected in either Trizol for RNA analysis or lysis buffer for western blotting analysis, or fixed in 10% v/v neutral buffered formalin for sectioning and immunostaining.

### Statistical analysis

Data are expressed as mean ± SEM. All experiments were performed with at least three replicate experiments and analysed using a Mann–Whitney U‐test or one‐way ANOVA in GraphPad Prism v. 6. MTT assay and tumour growth curve analysis used the two‐way ANOVA test. Fisher's exact test was used in tumour metastasis analysis. All statistical tests were two‐sided.

## Results

### ASCT2 expression is increased in prostate cancer

**Figure 1:**
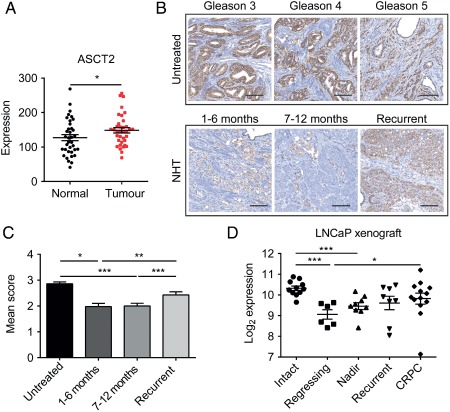
ASCT2 is androgen receptor‐regulated and expressed in prostate cancer patient samples and xenografts. (A) ASCT2 mRNA expression in matched prostate cancer samples compared to adjacent normal prostate from the TCGA dataset (data are mean ± SEM; paired t‐test; n = 36). (B) Representative images of ASCT2 protein expression in prostate cancer patient samples from Gleason grades 3, 4 and 5 and after neoadjuvant hormone therapy (NHT) treatment following an interval of 1–6 months, 7–12 months and in recurrent cancer; scale bar = 100 µm. (C) Immunohistochemical scoring of ASCT2 expression in patient cohorts before (n = 46) and after NHT treatment for 1–6 months (n = 54), 7–12 months (n = 76) and recurrent cancer (n = 32); data are mean ± SEM; Mann–Whitney U‐test. (D) Microarray analysis of ASCT2 mRNA expression from LNCaP xenograft tumours harvested from non‐castrated mice (intact; n = 10), post‐castration regressing tumours (regressing; n = 6), at PSA nadir after castration (nadir; n = 10), after recurrent prostate cancer had developed (recurrent; n = 6) and castration‐resistant prostate cancer (CRPC; n = 13); data are mean ± SEM; Mann–Whitney U‐test; ^*^p < 0.05, ^**^ p < 0.01, ^***^ p < 0.001

To determine whether ASCT2 expression is increased in prostate cancer compared to normal tissue, we compared patient‐matched *TCGA* mRNA expression data for normal prostate versus prostate cancer (Figure 1A). There was a significant increase in ASCT2 expression in tumour samples compared to normal (pairwise *t*‐test, *p =* 0.025), with 24/36 patients showing increased tumour ASCT2 levels compared to patient‐matched normal prostate tissue (χ^2^ test, *p =* 0.046). Analysis of Oncomine datasets showed significant increases in ASCT2 expression (*p <* 0.05) in seven prostate cancer datasets (Table 1). To determine ASCT2 protein levels, we performed immunohistochemical staining on a human prostate cancer tissue microarray (TMA; Figure 1B). At all stages of prostate cancer, ASCT2 protein was detected in the membrane of the prostate epithelial cells, but not in the cytoplasm (see supplementary material, Figure S1A). There were no significant differences in expression between Gleason grades in either the TMA or the TCGA dataset (see supplementary material, Figure S1B, C). Comparing untreated with patients undergoing neoadjuvant hormone therapy (NHT), a significant decrease in ASCT2 protein expression was observed during 1–6 and 7–12 months of treatment (*p =* 0.04; *p <* 0.001; Figure 1C). This supports previous data showing that ASCT2 expression is regulated by the androgen receptor 23. Interestingly, ASCT2 expression significantly increased in patients with recurrent prostate cancer (*p <* 0.001; Figure 1C), indicating either re‐activation of androgen receptor signalling or that other signalling pathways might up‐regulate ASCT2 expression upon disease recurrence. Analysis of an LNCaP xenograft model 24 at various stages precastration (intact), postcastration during regression, at the nadir of PSA, and in recurrent and castration‐resistant prostate cancer (CRPC) further confirmed this dynamic regulation of ASCT2 expression. ASCT2 expression decreased significantly post‐castration (regression and nadir), before increasing in recurrent and CRPC, where ASCT2 expression was no longer significantly different from intact ASCT2 levels (Figure 1D). These human and xenograft data suggest that ASCT2 levels may be important, both in the primary tumour and during tumour recurrence and CRPC.

**Table 1:** SLC1A5/ASCT2 expression in Oncomine datasets

Dataset	Fold change	*p*	PCa samples (*n*)
Su multi‐cancer	2.36	1.67E‐09	26
Singh prostate	2.106	3.24E‐04	52
Ramaswamy multi‐cancer	2.087	7.44E‐04	10
Wallace prostate	1.745	5.11E‐04	69
Magee prostate	1.518	0.018	8
Bittner multi‐cancer	1.459	3.64E‐12	59
Welsh prostate	1.399	7.00E‐03	25

### Inhibition of ASCT2 suppresses prostate cancer cell growth

**Figure 2:**
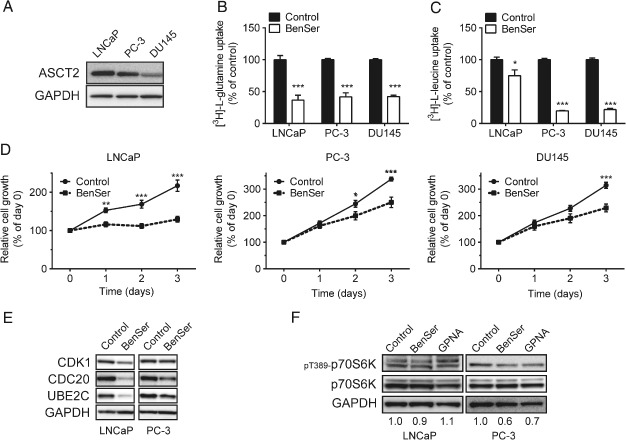
Inhibition of ASCT2‐mediated glutamine transport in prostate cancer cell lines. (A) ASCT2 protein was detected by western blotting in LNCaP, PC‐3 and DU145 cell lines. (B, C) Effect of BenSer (10 mm) on glutamine (B) and leucine (C) transport in LNCaP, PC‐3 and DU145 cell lines was assessed. (D) Cell growth of LNCaP, PC‐3 and DU145 was examined in the presence of BenSer. (E) CDK1, CDC20 and UBE2C expressions were detected by western blotting in LNCaP and PC‐3 cells in the presence of BenSer. (F) mTORC1 pathway activation (p‐p70S6K) was analysed in LNCaP and PC‐3 cells after BenSer or GPNA treatment after 2 h. (B, C) Data are mean ± SEM (n ≥ 3); Mann–Whitney U‐test was used to analyse data. (D) Data are mean ± SEM (n ≥ 3); two‐way ANOVA test was used to analyse data; ^*^p < 0.05, ^**^ p < 0.01, ^***^ p < 0.001

Analysis of ASCT2 protein showed high expression in both LNCaP and PC‐3 cells, with lower levels detected in DU145 cells (Figure 2A). Addition of the ASCT2 competitive inhibitor benzylserine (BenSer; see supplementary material, Figure S2A) 25 significantly reduced both glutamine and leucine uptake in LNCaP, PC‐3 and DU145 cells (Figure 2B, C). Leucine uptake inhibition occurs due to exchange of glutamine for leucine by l‐type amino acid transporter 1 (LAT1; SLC7A5), but may also be through direct LAT1 inhibition 15. Inhibition of androgen receptor signalling by bicalutamide also significantly reduced glutamine uptake in androgen‐sensitive LNCaP cells, but not androgen‐insensitive PC‐3 cells (see supplementary material, Figure S2B, C), confirming that AR signalling regulates ASCT2 levels 23 and ASCT2‐mediated glutamine transport. After 3 days of treatment with BenSer, cell growth was significantly decreased in LNCaP, PC‐3 and DU145 cells (Figure 2D). We have previously shown that LAT1 inhibition affects the cell cycle through E2F‐regulated cell cycle proteins CDK1, CDC20 and UBE2C 23. These three cell cycle‐regulatory proteins are also significantly increased in prostate cancer metastasis 23. CDK1, CDC20 and UBE2C exhibited decreased expression after BenSer treatment (Figure 2E).

### Inhibition of ASCT2 suppresses glutamine metabolism in prostate cancer

As leucine and glutamine are involved in the activation of mTORC1 signalling 14, 26, 27, we examined T389 phosphorylation of p70S6K, a downstream target of mTORC1. BenSer reduced the phosphorylation of p70S6K in PC‐3 cells, but not in LNCaP cells (Figure 2F). Utilizing another ASCT2 inhibitor, l‐γ‐glutamyl‐*p*‐nitroanilide (GPNA; see supplementary material, Figure S2D)^28^, confirmed that glutamine deprivation inhibits the mTORC1 pathway in PC‐3 cells, but not LNCaP cells (Figure 2F). These data suggest that mechanisms other than mTORC1 must also contribute to regulate cell growth in prostate cancer.

**Figure 3:**
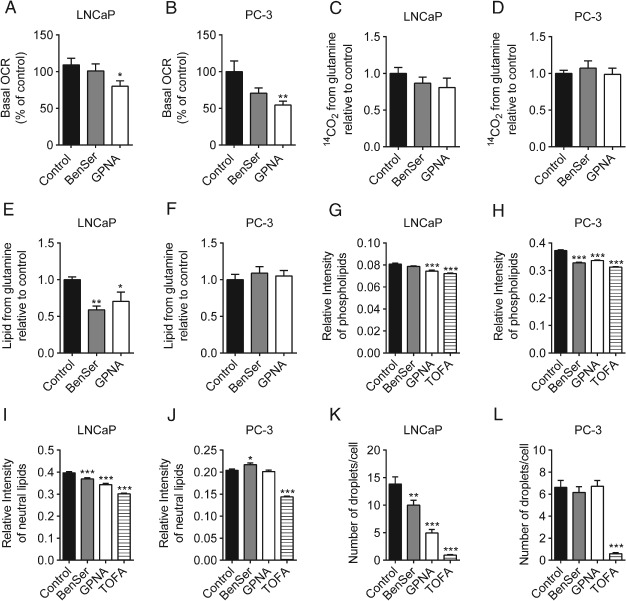
Inhibition of ASCT2 affects cell metabolism in LNCaP and PC‐3 cells. (A, B) Basal oxygen consumption rate (OCR) was analysed using a SeaHorse XF Analyser in LNCaP (A) and PC‐3 (B) cells pretreated with BenSer (10 mm) or GPNA (1 mm). (C, D) ^14^CO_2_ generated from glutamine in LNCaP (C) and PC‐3 (D) cells was determined after treatment with BenSer (10 mm) or GPNA (1 mm). (E, F) Lipid generated from glutamine was analysed in LNCaP (E) and PC‐3 (F) cells after treatment with BenSer (10 mm) or GPNA (1 mm). (G–J) Mean fluorescence intensities of cellular phospholipids (G, H) and neutral lipids (I, J) were measured in LNCaP (G, I) and PC‐3 cells (H, J) after treatment for 24 h with BenSer (10 mm), GPNA (1 mm) or TOFA (10 µm). (K, L) Lipid droplets were measured in LNCaP (K) and PC‐3 (L) cells after treatment with BenSer, GPNA or TOFA. (A–F) Data are mean ± SEM (n ≥ 3); one‐way ANOVA was used to analyse data. (G–L) Data are mean ± SEM (n ≈ 300 cells); one‐way ANOVA was used to analyse data; ^*^p < 0.05, ^**^ p < 0.01, ^***^ p < 0.001

The majority of intracellular glutamine is converted into glutamate, which can be utilized by the TCA cycle for ATP and fatty acid production, or exported from the cell by xCT (SLC7A11) in exchange for cystine, thereby contributing to glutathione production and protection against oxidative stress. Alternatively, glutamine acts as a nitrogen and carbon donor for macromolecular synthesis, including nucleotides and non‐essential amino acids/proteins. We therefore set out to determine how changes in intracellular glutamine affect these downstream pathways. Using the Seahorse flux analyser, BenSer or GPNA treatment resulted in a reduction in basal oxygen consumption rates (OCRs) compared to control in LNCaP and PC‐3 cells (see supplementary material, Figure S2E, F). GPNA, but not BenSer, significantly reduced basal OCR in both LNCaP and PC‐3 cells (Figure 3A, B). However, this reduction in OCR did not appear to result directly from glutamine usage, as there was no significant reduction in oxidized ^14^C‐labelled glutamine in either cell line after BenSer or GPNA (Figure 3C, D), despite lower ^14^C‐glutamine uptake (see supplementary material, Figure S2G, H). Lipid synthesis from glutamine, however, was significantly decreased in LNCaP cells (Figure 3E), but not in PC‐3 cells (Figure 3F). These data suggest that LNCaP cells directly utilize glutamine for fatty acid synthesis, while PC‐3 cells may utilize glutamine for other pathways, including exchange for leucine (Figure 2C) to activate mTORC1 signalling (Figure 2F), which indirectly affects mitochondrial respiration.

To further investigate lipid homeostasis, we measured the levels of cellular phospholipids and neutral lipids. Lipid content analysis of LNCaP cells revealed that GPNA significantly reduced the cellular level of phospholipids (Figure 3G) and BenSer and GPNA reduced the neutral lipids (Figure 3I) as well as lipid droplet number (Figure 3K). Notably, the magnitude of inhibition by BenSer and GPNA was similar to thast of 5‐(tetradecyloxy)‐2‐furoic acid (TOFA), which inhibits the rate‐limiting enzyme of fatty acid synthesis, acetyl‐CoA carboxylase (Figure 3G, I, K). A significant decrease of phospholipids was also observed in PC‐3 cells (Figure 3H); however, there were no significant decreases in neutral lipids (Figure 3J) or lipid droplet numbers (Figure 3L). Taken together, pharmacological inhibition of ASCT2 provoked cell line‐specific effects on the flux of glutamine as a fuel source, yet ultimately led to reduced OCR and cellular lipid levels in both prostate cancer cell lines. However, neither BenSer nor GPNA led to increased levels of reactive oxygen species (ROS; see supplementary material, Figure S2I).

### Global effects of BenSer or GPNA inhibition in PC‐3 cells

**Figure 4:**
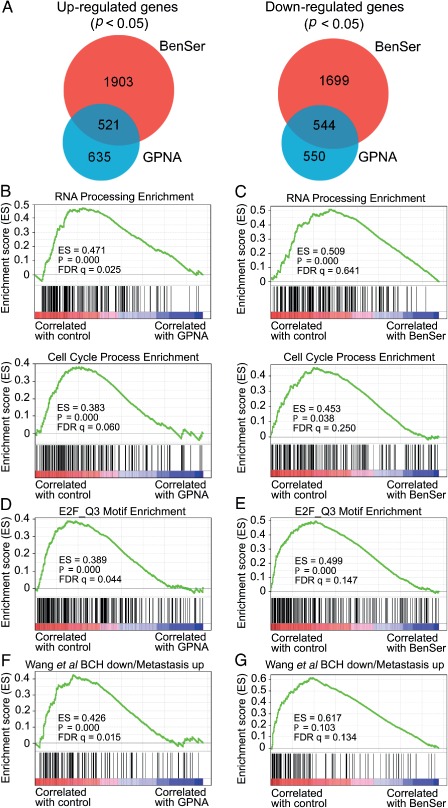
RNA‐seq analysis of PC‐3 cells treated with BenSer or GPNA. (A) Venn diagram of genes up‐ or down‐regulated in both BenSer‐ and GPNA‐treated groups. (B, C) Gene set enrichment analysis (GSEA) plot of Gene Ontology categories RNA Processing and Cell Cycle Process in control versus GPNA (1 mm; B) or BenSer (10 mm; C) treatment. (D, E) GSEA plot of E2F transcription factor motif gene set in control versus GPNA (1 mm; D) or BenSer (10 mm; E) treatment. (F, G) GSEA plot of BCH down‐regulated gene set (from 23) in control versus GPNA (1 mm; F) or BenSer (10 mm; G) treatment groups

To determine the global effects of ASCT2 inhibition, we used next‐generation sequencing to determine mRNA expression changes in PC‐3 cells treated with BenSer or GPNA for 48 h. Analysis of genes significantly (*p <* 0.05) up‐ or down‐regulated by GPNA (see supplementary material, Table S1) compared to control showed a substantial overlap (up‐regulated, 45.1%; down‐regulated, 49.7%) with BenSer gene expression, highlighting common targets of ASCT2 (Figure 4A). BenSer appears to have additional non‐ASCT2‐mediated affects, with more genes showing significant up‐ and down‐regulation (see supplementary material, Table S2) compared to GPNA (Figure 4A). This is not surprising, since BenSer has been shown to inhibit both ASCT2 and LAT1 function in oocytes 18.

Gene set enrichment analysis (GSEA) using Gene Ontology categories showed significant enrichment in control compared to GPNA for RNA and DNA processing and metabolism, as well as cell cycle processes (Figure 4B; see also supplementary material, Table S3). Similar categories were also enriched in control compared to BenSer (Figure 4C; see also supplementary material, Table S4). Analysis of motif enrichment by GSEA showed a significant association with E2F transcription factor motifs for control compared to either GPNA or BenSer (Figure 4D, E; see also supplementary material, Tables S5, S6). We have previously shown that inhibition of leucine uptake in LNCaP cells shows similar E2F‐mediated cell cycle inhibition, with a subset of 122 genes down‐regulated by the leucine uptake inhibitor BCH 23. Analysis of this 122‐gene signature in control versus GPNA and BenSer showed enrichment in the control group (Figure 4F, G), suggesting that there is a common mechanism regulating gene expression after leucine starvation and glutamine starvation in prostate cancer.

Amino acid starvation leads to up‐regulation of the stress‐response transcription factor ATF4, which in turn leads to increased expression of diverse amino acid transporters, including ASCT2 and LAT1 23. ATF4 was the first (*p =* 1.10E‐11) and sixteenth (*p =* 4.21E‐21) most significantly up‐regulated gene in GPNA‐ or BenSer‐treated cells, respectively (see supplementary material, Tables S1, S2), which was verified by RT–qPCR (see supplementary material, Figure S3A, B). ATF4 has previously been shown to directly transcriptionally regulate the expression of transporters including ASCT2, ASCT1, xCT and SLC3A2 23, 29. Analysis of gene sets up‐regulated after GPNA inhibition showed significant enrichment for Gene Ontology categories, including amino acid transport (see supplementary material, Figure S3C), with significant up‐regulation of transporters, including SLC1A5 (ASCT2, *p =* 0.020), SLC1A4 (ASCT1; *p =* 7.72E‐5), SLC7A11 (xCT; *p =* 7.02E‐8), SLC38A1 (SNAT1; *p =* 3.51E‐5), SLC7A8 (LAT2; *p =* 5.63E‐5), SLC3A2 (4F2hc; *p =* 0.041) and SLC3A1 (*p =* 0.005). Another critical gene significantly increased by both GPNA (*p =* 0.009) and BenSer (*p =* 0.0002) was EIF2AK3 (PERK), which can phosphorylate eIF2α, leading to global protein synthesis suppression. A set of genes that belong to eukaryotic translation initiation factor are significantly decreased after GPNA treatment, including EIF3B (*p =* 0.0003), EIF2A (*p =* 0.003), EIF4H (*p =* 0.005), EIF5A (*p =* 0.009), EIF2B3 (*p =* 0.034), EIF4G1 (*p =* 0.039), EIF2B4 (*p =* 0.040) and EIF2B5 (*p =* 0.048).

### Knockdown of ASCT2 suppresses growth in prostate cancer cells

**Figure 5:**
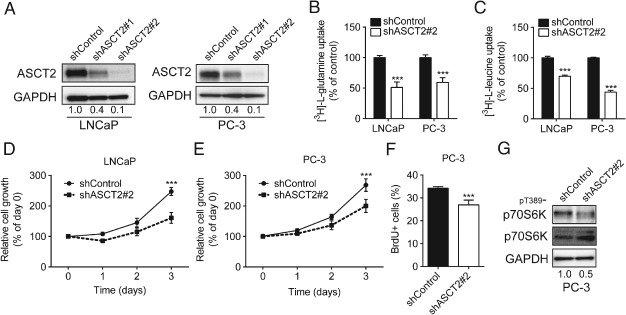
ASCT2 is required for prostate cancer cell proliferation. (A) Analysis of ASCT2 protein after shRNA knockdown in LNCAP or PC‐3 cells using shASCT2#1 or shASCT2#2. (B, C) Analysis of glutamine (B) or leucine (C) uptake in LNCaP or PC‐3 cells after shASCT2 knockdown. (D, E) LNCaP (D) and PC‐3 (E) cell growth after shASCT2#2 knockdown was analysed by MTT assay. (F, G) Cell cycle progression (BrdU incorporation; F) and mTORC1 pathway activation (p‐p70S6K; G) was analysed after shASCT2#2 knockdown in PC‐3 cells. (B, C, F) Data are mean ± SEM (n ≥ 3); Mann–Whitney U‐test was used to analyse data. (D, E) Data are mean ± SEM (n ≥ 3); significance was assessed using two‐way ANOVA; ^*^p < 0.05, ^**^ p < 0.01, ^***^ p < 0.001

We used lentiviral shRNA‐mediated knockdown of ASCT2 to substantiate the direct ASCT2 effects of BenSer and GPNA in LNCaP and PC‐3 cells. Two different shRNAs decreased ASCT2 expression and glutamine transport in LNCaP and PC‐3 cells compared to a non‐targeting shRNA control (Figure 5A, B; see also supplementary material, Figure S3D). Inhibition using shASCT2#2 also significantly decreased leucine uptake in both LNCaP and PC‐3 cells (Figure 5C), suggesting that ASCT2‐transported glutamine is utilized for leucine uptake.

LNCaP and PC‐3 cells expressing shASCT2#2 showed a significant decrease in cell viability compared to shControl cells (Figure 5D, E). Analysis of BrdU incorporation (Figure 5F) and apoptosis (see supplementary material, Figure S3E) in shASCT2#2‐expressing PC‐3 cells confirmed that ASCT2 knockdown inhibits the cell cycle rather than apoptosis. Similar results for glutamine uptake, BrdU incorporation and apoptosis were also observed in shASCT2#1 PC‐3 cells (see supplementary material, Figure S3D, F, G). Finally, we examined mTORC1 pathway activation by western blotting, showing reduced phosphorylation of p70S6K in PC‐3 cells expressing shASCT2#2 (Figure 5G).

### Knockdown of ASCT2 suppresses tumour growth in prostate cancer xenografts

**Figure 6:**
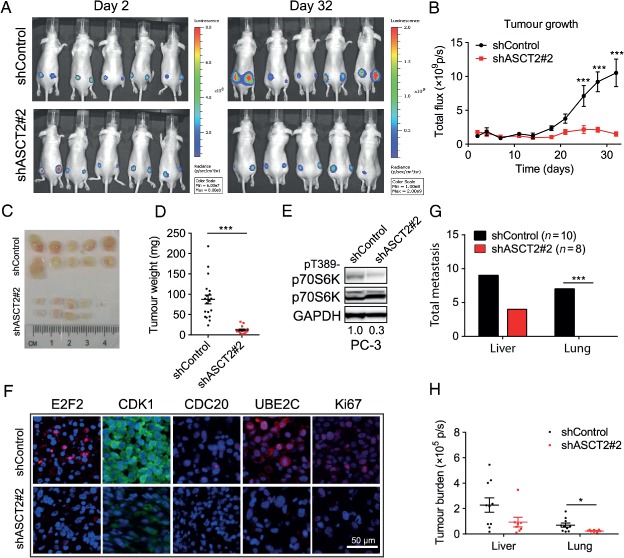
ASCT2 is required for tumour growth in vivo. (A) PC‐3‐luc cells stably expressing shControl or shASCT2#2 were injected subcutaneously into the right and left dorsal flanks of male nude mice; bioluminescent images are shown at days 2 and 32. (B) Tumour growth was measured twice weekly by bioluminescence in shControl (n = 10) and shASCT2 (n = 8) mice; data are mean ± SEM; significance was assessed using two‐way ANOVA; ^***^ < 0.001. (C, D) Tumours (shControl, n = 20; shASCT2#2, n = 15) were harvested after 32 days, imaged (C) and weighed (D). (E) Phosphorylated p70S6K was measured after shASCT2 knockdown in PC‐3 xenograft. (F) Sections from shControl and shASCT2 tumours were stained for CDK1, CDC20, UBE2C and Ki67 expression; representative images; scale bar = 100 µm. (G, H) Number (G) and size (H) of spontaneous metastases in liver and lung were measured by bioluminescence. (B) Data are mean ± SEM; significance was assessed using two‐way ANOVA. (D, H) Data are mean ± SEM; Mann–Whitney U‐test was used to analyse data. (G) Number of spontaneous metastases at day 32 in shControl and shASCT2 mice was assessed using two‐tailed Fisher exact test; ^*^p < 0.05, ^**^ p < 0.01, ^***^ p < 0.001

To determine whether ASCT2 function is critical for tumour growth *in vivo*, PC‐3 cells expressing shControl or shASCT2#2 were transduced with a lentiviral vector co‐expressing eGFP and firefly luciferase 30. Cells were enriched to high purity on a narrow band of high GFP expression, resulting in similar GFP/luciferase expression in each shControl and shASCT2 cell line (see supplementary material, Figure S4A). PC‐3–luc cells were subcutaneously injected into nude mice, with similar luciferase expression in shControl and shASCT2 tumours confirmed after 48 h (Figure 6A). Bioluminescence was analysed twice weekly for 32 days, showing a significant decrease in shASCT2 tumour size by day 25 (Figure 6B).

Mice were euthanized after 32 days, due to the size of the shControl tumours. The tumours were isolated, photographed and weighed, with shControl tumours being significantly larger than shASCT2 tumours (Figure 6C, D; *p <* 0.001). Western blots of xenograft tumours showed that phosphorylation of p70S6K was decreased in shASCT2 tumours compared to shControl tumours (Figure 6E). Analysis of E2F2, CDK1, CDC20, UBE2C and the proliferation biomarker Ki67 in xenograft sections showed consistently lower levels in the shASCT2 tumours (Figure 6F). There were no changes in cleaved caspase 3 levels (see supplementary material, Figure S4B).

Micrometastases were detected by isolation and *ex vivo* bioluminescence analysis of organs immediately after euthanasia (see supplementary material, Figure S4C). Analysis of shControl mice showed that nine of 10 had metastases in the liver and four of 10 in the lung, while shASCT2 mice showed that four of nine had metastases in the liver, with no lung metastases (*p =* 0.004; Figure 6G). Metastatic bioluminescence burden in the liver and lung also showed a significant decrease in shASCT2 xenografted mice (Figure 6H).

## Discussion

ASCT2 can be regulated by multiple transcription factors, including Myc 6, Rb/E2F 31, androgen receptor 23 and ATF4 21, 23. This permits similar ASCT2 protein expression in both androgen‐dependent (LNCaP) and androgen‐independent (PC‐3) prostate cancer cell lines, and facilitates sufficient glutamine for cell growth. Our patient and LNCaP xenograft data confirmed that androgen receptor regulation of ASCT2 contributes to its expression in untreated/primary cancer and that under androgen‐deprivation therapy, ASCT2 levels decrease. Similar to other androgen receptor‐ and ATF4‐regulated genes 23, re‐expression of ASCT2 was observed in recurrent disease. Importantly, ATF4 levels are significantly increased in androgen‐independent prostate cancer metastasis compared to primary cancer (2.25‐fold; *p =* 3.82E‐7) 23, suggesting that activation of ATF4 transcriptional targets is important in either metastasis or maintenance of the metastatic tumour. Interestingly, ASCT2 knockdown tumours showed decreased metastasis in the liver and lung, although this was confounded by the smaller primary tumours.

Blocking ASCT2 using either chemical or genetic means reduced cell proliferation and cell cycle in both LNCaP and PC‐3 cell lines. This coincided not only with reduced glutamine levels but also with a reduction in the essential amino acid leucine, likely through LAT1‐mediated exchange for leucine. Although knockdown of ASCT2 resulted in similar glutamine uptake inhibition in LNCaP and PC‐3 cells, leucine uptake was inhibited at a higher level in PC‐3 cells, coincident with the higher LAT1 expression compared to LNCaP cells 32, further supporting the ASCT2–LAT1 exchange hypothesis, which subsequently leads to altered mTORC1 signalling 15, 33. ASCT2 inhibition did not directly alter glutamine metabolism in PC‐3 cells, suggesting that the effects on basal OCR may be due to combined glutamine/leucine inhibition and subsequent shut‐down of energy‐consuming processes, such as protein, lipid, RNA and DNA synthesis. Previous studies have shown that mTORC1 controls mitochondrial activity through a 4EBP‐dependent translation regulation 34 and mitochondrial gene expression 35. This mechanism is further supported by the GPNA‐mediated down‐regulation of GO categories for RNA and DNA Processing and Mitochondrial Genesets in PC‐3 cells. Analysis of glutamine metabolism in LNCaP cells, however, showed that inhibition of ASCT2 suppresses basal oxygen consumption, as well as TCA cycle glutaminolysis through complete oxidation and conversion to lipids. LNCaP cells have low expression of LAT1 and high expression of the leucine uniporter LAT3, and thereby are not as reliant on glutamine exchange to maintain leucine levels. They appear more reliant on glutamine anaplerosis than PC‐3 cells.

Analysis of gene expression in PC‐3 cells treated with BenSer or GPNA showed an adaptive response to glutamine deprivation through ATF4‐mediated transcription, resulting in reduced cell cycle and RNA processing, and up‐regulation of amino acid transporters in an attempt to restore glutamine levels. Previous studies have suggested that targeting this ATF4‐mediated stress response may provide an effective cancer therapy 36. However, in glioblastoma, the ATF4 response to glutamine deprivation directs apoptosis through NOXA and PUMA 37. Unlike glioblastoma, we did not observe an increase in apoptosis in PC‐3 cells after ASCT2 inhibition. These chemicals, or ASCT2 shRNA knockdown, do not induce complete glutamine deprivation, suggesting that perhaps the magnitude of glutamine deprivation, and subsequent ATF4 activation, may be critical in driving cell fate decisions for survival or apoptosis.

High expression of ASCT2 has previously been reported in the normal prostate 17 and many rapidly dividing normal cells rely on ASCT2 expression for glutamine uptake. Despite this important function in normal cells, the ASCT2 knockout mice do not show any obvious abnormalities in growth or survival, making ASCT2 an attractive therapeutic target 40. Compared to the immunosuppressive effects of directly inhibiting mTORC1, the ASCT2 knockout mice have a relatively mild immune phenotype involving Th1 and Th17 lineage differentiation 40. Despite this mild phenotype, it will still be important to monitor immune cells and effects on other normal cells when developing ASCT2‐targeted therapies.

Our *in vivo* knockdown data support the development of ASCT2 as a therapeutic target in prostate cancer. Since ASCT2 is androgen‐regulated, one could envisage targeting ASCT2 in either primary or advanced prostate cancer. Importantly, our data showed that targeting ASCT2 not only inhibited known pathways, such as mTORC1, but also regulated the critical metastasis‐expressed, E2F‐regulated, cell cycle genes *CDK1*, *CDC20* and *UBE2C*, thereby resulting in a two‐pronged block of cell division. This suggests that ASCT2‐targeted therapies may be particularly effective in advanced castration‐resistant prostate cancer. However, current ASCT2 inhibitors, such as BenSer 38, GPNA 28 and dithiazoles 39, have high effective concentrations, rendering them ineffective as putative therapeutics. The development of new inhibitors in the low μm or nm range would be required for further preclinical testing. This is one of the major limitations of this current study. Additionally, once a drug is developed, it would be important to determine an appropriate clinical population and setting that may benefit from ASCT2 targeting, using preclinical models such as patient‐derived xenografts or explants. Further limitations surround the potential up‐regulation of other glutamine transporters by adaptive mechanisms such as ATF4 transcription; this will need to be monitored for potential compensation mechanisms in tumours. Finally, while glutamine is clearly an important amino acid transported by ASCT2, it remains to be determined what downstream effects are caused by inhibition of other ASCT2‐transported amino acids, such as alanine, serine, cysteine, threonine or asparagine.

In summary, we have shown that ASCT2 is the key glutamine transporter that regulates prostate cancer proliferation and metabolism. We have identified critical cell cycle and metabolic pathways, which may provide new avenues for therapeutic intervention in prostate cancer.

## Author contributions

Conception and design, JH and QW; development of methodology, JH, QW, CGB and WR; acquisition of data, QW, RH, AJH, MvG, MG, LF, JJLW, SB, MS, RN, CM, NP and NO; analysis and interpretation of data, JH, QW, RH, AJH, MCS, DG, WR, LF, ML, CCN and JEJR; writing, review and/or revision of the manuscript, JH, QW, JEJR and CGB; and study supervision, JH.

**Supplementary material on the internet**

The following supplementary material may be found in the online version of this article:

**Supplementary materials and methods**

**Figure S1.** ASCT2 IHC scoring and Gleason expression.

**Figure S2.** Inhibition of glutamine uptake and metabolism.

**Figure S3.** ATF4 activation and ASCT2 shRNA inhibition.

**Figure S4.** shASCT2 in vivo apoptosis and metastasis.

**Table S1.** Genes upregulated and downregulated by GPNA.

**Table S2.** Genes upregulated and downregulated by BenSer.

**Table S3.** Gene ontology upregulated in control vs GPNA.

**Table S4.** Gene ontology upregulated in control vs BenSer.

**Table S5.** Motif upregulated in control vs GPNA.

**Table S6.** Motif upregulated in control vs BenSer.

## Supporting information

AppendixS1. Supplementary Information

FigureS1. A, ASCT2 protein expression was detected on the cell membrane in patient samples. Representative images of each staining score are shown. Scale bar is 50 urn. B, scoring of ASCT2 protein expression in patient cohort with different Gleason grades. C, ASCT2 mRNA expression in the TCGA patient cohort with different Gleason scores. B, BPH, n = 36; Gleason 3,n = 59; Gleason 4, n = 44; Gleason 5, n = 7. Data are the mean ± SEM. Mann‐Whitney U‐test was used to analyze data. C, Gleason score 6, n = 11; Gleason score 7, n = 113; Gleason score 8, n = 12; Gleason score 9, n = 19. Data are the mean ± SEM. Mann‐Whitney U‐test was used to analyze data.

A, glutamine uptake was assessed at a range of BenSer concentrations. Band C, glutamine uptake was assessed in the presence of bicalutamide +/‐ BenSer (B) or DHT +/BenSer (C) in LNCaP and PC‐3. 0, glutamine uptake was assessed in the presence of GPNA (1 mM) in PC‐3 cells.E and F, oxygen consumption rate (OCR) was assessed on a SeaHorse XF Analyzer in LNCaP (E) and PC‐3 (F) cells pre‐treated with BenSer (10 mM) or GPNA (1 mM), followed by addition of oligomycin, FCCP, or rotenone and antimycin. G and H, glutamine uptake was decreased in LNCaP and PC‐3 cells in the presence of BenSer or GPNA. I, oxidative stress was measured in PC‐3 cells using CellRox reagent in the presence of BenSer (10 mM), GPNA (1 mM) or positive control TBHP (250 ˜ M). A‐H, data are the mean ± SEM (n = 3). 0, Mann‐Whitney U‐test was used to analyze data. B, C, E, F , one‐way ANOVA test was used to analyze data. ^*^, P < 0.05; ^**^, P < 0.01; ^***^, P < 0.001. n.s, no significance.

A, ATF4 mRNA exprssion was detected by qRT‐PCR in PC‐3 cells in the presence of BenSer or GPNA. B, PCR products are examined in a agarose gel electrophoresis. C, gene set enrichment analysis (GSEA) plot Gene Ontology categories Amino Acid Transport in control versus GPNA group. 0, glutamine uptake was assessed in shControl and shASCT2#1 expressing PC‐3 cells. E, annexin V staining was used to detect apoptosis in PC‐3 cells expressing shControl and shASCT2#2. F, cell cycle phase was determined using BrdU incorporation assay in PC‐3 cells expressing shControl and shASCT2#1. G, annexin V staining was used to detect apoptosis in PC‐3 cells expressing shControl and shASCT2#1. A, one‐way ANOVA test was performed. O‐G, data represent mean ± SEM, n = 3. Mann‐Whitney U test was performed. ^*^, P < 0.05; ^**^, P < 0.01; ^***^, P < 0.001.

**Figure**S4. A, PC‐3 cells expressing shControl or shASCT2 were transduced with GFP‐2A‐luciferase expressing construct and sorted for high GFP expression by FACS. B, cleaved caspase 3 expression in shControl and shASCT2. C, spontaneous metastatic PC‐3‐luc cells expressing shControl or shASCT2 were detected in the liver and lungs of mice bearing subcutaneous tumors.

TableS1 Genes upregulated and downregulated by GPNA

TableS2 Genes upregulated and downregulated by BenSer

TableS3 GSEA gene ontology upregulated gene sets in control vs GPNA treated PC‐3 cells (Top 50).

TableS4 GSEA gene ontology upregulated gene sets in control vs benzylserine treated PC‐3 cells (Top 50).

TableS5 GSEA motif upregulated gene sets in control vs GPNA treated PC‐3 cells (Top 50).

TableS6 GSEA motif upregulated gene sets in control vs benzylserine treated PC‐3 cells (Top 50).
