# Supplementary material for: Targeting ASCT2‐mediated glutamine uptake blocks prostate cancer growth and tumour development
Source: J Pathol. 2015 Apr 7;236(3):278–89. doi: 10.1002/path.4518 (PMC4973854; doi:10.1002/path.4518)
Supplement: Supplementary file 1 — AppendixS1. Supplementary Information [file PATH-236-278-s001.docx]

**Supplementary Information**

**Supplementary methods and materials:**

**Antibodies**

Antibodies used in this study were against ASCT2, pT389-p70S6K, p70S6K, cleaved caspase 3 (Cell Signaling Technology), UBE2C (Boston Biochem, Cambridge, MA), E2F2 (Santa Cruz), Ki67, CDC20, CDK1 and glyceraldehyde-3-phosphate dehydrogenase (GAPDH; Abcam). Horseradish peroxidase–conjugated donkey anti-mouse IgG and donkey anti-rabbit IgG were used as secondary antibodies (Millipore).

**Uptake assay**

Cells were cultured in 6-well plates in RPMI media. After collecting and counting, cells (3×10^4^/well) were incubated with 0.3 µCi [^3^H]-L-leucine (200 nM; PerkinElmer) in leucine-free RPMI media for 15 min at 37°C in the presence or absence of each inhibitor. For the glutamine uptake assay, cells (1×10^5^/well) were incubated with [^3^H]-L-glutamine (400 nM; PerkinElmer) in minimum essential media (Life Technologies) for 15 min at 37°C in the presence or absence of each inhibitor. Cells were collected and transferred to filter paper using a 96-well plate harvester (Wallac PerkinElmer), dried and exposed to scintillation fluid. Counts are measured using a liquid scintillation counter (PerkinElmer). For bicalutamide or DHT treatment, cells were pre-treated with either DMSO, bicalutamide (25 µM) or DHT (10 nM) for 48 h, followed by the glutamine uptake assay.

**BrdU analysis**

Cells (2×10^5^ per well) were seeded in 6-well plates and allowed to adhere overnight. After serum starvation, cells were incubated in the fresh RPMI media for 22 h, followed by addition of BrdU (150 µg/mL) for another 2 h. Cells were detached using Tryple (Life Technologies), fixed and stained using the APC-BrdU Flow Kits (BD, Biosciences). The BrdU antibody was diluted 1:50. Nuclei were counter-stained with 7-AAD. Both cell cycle and BrdU analysis was performed using a BD Canto II flow cytometer and FlowJo software (Tree Star Inc.).

**Annexin V assay**

Cells (2×10^5^ per well) were seeded in 6-well plates, allowed to adhere overnight. Positive control cells were irradiated in a UV Stratalinker 2400 (Stratagene) with a 400,000 μJ dosage and incubated in fresh media for 16 h. Cells were detached using Tryple and resuspended in 1 mL of binding buffer (HEPES–buffered PBS supplemented with 2.5 mM calcium chloride) containing anti-annexin V-APC (BD) and incubated for 15 min in the dark at room temperature. PI solution (20 μg/mL) was added, and the cells were analyzed using a BD Canto II flow cytometer and FlowJo software (Tree Star Inc.).

**SDS-PAGE and Western blotting**

Cells (2×10^5^ per well) were seeded in 6-well plates, allowed to adhere overnight. Cells were lyzed by the addition of lysis buffer (200 μL; 20 mM Tris–HCl, 150 mM NaCl, 1% (v/v)Triton X-100, 0.5% (w/v) Na deoxycholate, 0.1% (w/v) SDS) with protease inhibitor Cocktail III (Bioprocessing Biochemical, California) and 1 mM Na_3_VO_4_ (Sigma). Equal protein (micro-BCA method; Pierce, IL) was loaded on 4–12% gradient gels (Life Technologies), electrophoresis performed and transferred to PVDF membrane. The membrane was blocked with 2.5% (w/v) BSA in PBS­-Tween20, and incubated with the primary and secondary antibodies. The secondary HRP-labelled antibodies were detected using enhanced chemiluminescence reagents (Pierce) on a Kodak Imager (Kodak). The results were analyzed by densitometry using ImageJ Software.

**LNCaP castration xenograft experiment**

LNCaP xenograft tumors were grown in athymic nude mice at two sites as modified from a previously reported method [1,2]. PSA (prostate-specific antigen) serum levels were measured weekly from blood samples collected from tail vein incisions. Animals were castrated when serum PSA was > 100 ng/mL or the tumor was ~1.5 cm diameter (approximately 8 weeks after injection with LNCaP cells) [1]. The animals were sacrificed, tumors collected and mRNA expression analyzed by microarray as detailed previously [1,2]. Data were analyzed from the GEO Database GSE44319 for ASCT2 (Probe P_126177) expression.

**Immunofluorescence staining**

PC-3-luc xenografts from a single experiment were sectioned. For immunofluorescence staining, sections were boiled in sodium citrate buffer (10 mM, pH=6) for 20 min, followed by a rinse in distilled water. After incubation with 5% (v/v) normal goat serum for 30 min, sections were stained for Ki67, CDK1, UBE2C, CDC20 or E2F2 (all antibodies 1:100) overnight at 4°C. The cells were washed in PBS before addition of a goat anti-rabbit Alexa 594 (1:1000; Life Technologies) for CDC20, UBE2C and Ki67, goat anti-mouse Alexa 488 (1:1000, Life Technologies) for CDK1 and goat anti-mouse Alexa 594 (1:1000, Life Technologies) for E2F2 for 2 h at room temperature. After washing with PBS, slides were mounted by ProLong Gold anti-fade reagent with DAPI (Life Technologies). Staining results were imaged by Leica DM6000B (Leica).

**Measurement of oxygen consumption**

Cellular oxygen consumption rate (OCR) was measured with a Seahorse XF analyzer (Seahorse Bioscience, MA) according to manufacturer’s instructions. Briefly, cells (2×10^4^ cells/well for PC-3 or 2.5×10^4^ cells/well for LNCaP) were seeded in a Seahorse XF 24-well assay plate in full growth medium. After overnight attachment, the medium was washed and replaced with pre-warmed running medium (consisting of non-buffered DMEM (Sigma-Aldrich, MO) supplemented with 1 mM sodium pyruvate and 10 mM glucose, pH 7.4) containing either 10 mM BenSer or 1 mM GPNA. Plates were incubated for 60 min in a non-CO_2_ incubator at 37°C before three basal measurements were undertaken determining oxygen and proton concentration in the medium. Then the ATP synthase inhibitor oligomycin (1 µg/mL), Carbonyl cyanide-*4*-(trifluoromethoxy)phenylhydrazone (FCCP; 0.3 µM) and the complex III inhibitor rotenone/antimycin A (1 µM) were injected serially, with three further measurements after each addition. Seahorse plates had all media removed, without disrupting the cell monolayer, were lyzed, and then frozen at -20°C, immediately after the conclusion of the assay. Total protein in each well was determined using bicinchoninic acid (BCA) assay (Pierce, IL), according to manufacturer’s instructions. All OCR values were normalized to total protein in each well.

**Measurement of lipid synthesis and oxidation**

LNCaP and PC-3 cells were seeded in 6-well plates with 2×10^5^ cells/well and treated with BenSer or GPNA for 20 h. Media was then changed to 500 µL glutamine-free DMEM media with addition of 2% BSA, 0.5 mM oleate, 1 mM carnitine, 0.5 µCi/ml [^14^C]-L-glutamine, and 2 mM L-glutamine. Cells were incubated at 37°C for 4 h. Subsequently, the media was transferred to a glass scintillation vial housing both a microfuge tube containing 400 µL sodium hydroxide and 500 μL of 1 M PCA. The vial was shaken for 2 h. The sodium hydroxide was transferred into a scintillation vial, containing 3 mL of scintillation buffer (Ultima gold, PerkinElmer). Radiation was measured by scintillation counter (1900CA Tri-Carb Liquid Scintillation Analyzer, Packard). Cells were rinsed in PBS and lipids extracted in chloroform:methanol (2:1 v:v). Following centrifugation (1,000 g for 10 min), the organic phase was evaporated to dryness under nitrogen gas at 40°C, lipids resuspended in 30 μL of 100% ethanol then transferred to a scintillation vial containing 3 mL of scintillation buffer to determine ^14^C activity. Cell lysates were collected to determine the total protein using BCA assay (Pierce, IL).

**Lipid content analysis**

LNCaP (4000 cells/well) and PC-3 cells (2000 cells/well) were seeded in 96-well plates for 48 h and treated with BenSer (10 mM), GPNA (1 mM) or TOFA (10μM; Sigma-Aldrich) for 24 h. As described previously [3], cells were fixed and stained with DAPI (1 μg/ml, Sigma-Aldrich) and Nile Red (0.25 μg/ml, Sigma-Aldrich) in PBS for 20 min in the dark. Cells were imaged on an IN Cell Analyzer 2200 (GE Healthcare) with a 10× magnification, and fluorescence of neutral lipids and phospholipids were measured in the Cy3 and Cy5 channels, respectively. Image segmentation and quantitation of mean cellular fluorescence intensities of neutral lipids and phospholipids and lipid droplet count of 300 cells were performed with CellProfiler software (Broad Institute).

**ROS assessment**

Cells were treated with BenSer (10 mM), GPNA (1 mM) or tert-butyl hydroperoxide (TBHP; 250 µM) as positive control for 30 min at 37°C, followed by incubation with CellRox Deep Red reagent (500 nM; Life Technologies) for 30 min. The cells were analyzed using a BD Canto II flow cytometer and FlowJo software.

**Quantitative Real Time- PCR (qRT-PCR)**

For qRT-PCR of *ATF4* transcripts in PC3 cells, total RNA samples were treated with DNAse I, amplification grade (Invitrogen). cDNA was generated using SuperScript® III First-Strand Synthesis System (Invitrogen), according to the manufacturers’ instructions. PCR was performed using Platinum^®^ *Taq* polymerase (Invitrogen) with the following primer set: hATF4-F, 5′ -CTTAAGCCATGGCGCTTCT-3′ and hATF4-R, 5′-GAGAAGGCATCCTCC TTGCT -3′. Expression of human *B2M1* (*hB2M1*) was used to normalise for cDNA input. The following primer set was used hB2M1-F,5′-TGCCGTGTGAACCATGTGAC-3′ and hB2M1-R, 5′-CGGCATCTTCAAACCTCCAT-3′

For each sample, equal amount of amplified products (10 μg) were used for agarose gel electrophoresis. Quantitative RT-PCR was performed using the CFX96^(TM)^ Real Time PCR Machine (Bio-Rad). Reactions were performed in 20 μl volumes containing 1×IQ SyberGreen supermix and 0.3 μM of each ATF4 forward and reverse primers (hATF4-2F, 5’- TCAAACCTCATGGGTTCTCC -3’; hATF4-2R. 5’- GAGAAGGCATCCTCCTTGCT -3’). Normalisation of cDNA input was performed for *B2M1* using the same primers for qRT-PCR. Cycling conditions were: 95°C for 6 min followed by 95°C for 30 sec, primer-specific annealing temperature for 30 sec, and extension at 72°C for 30 sec. Fold changes were calculated using the ∆∆CT method.

**Lentiviral shRNA expression**

ASCT2 shRNA lentiviral preparation was performed as previously described [4]. Briefly, pLKO.1 plasmids containing short hairpin RNA (shRNA) against ASCT2 or a non-targeting *Arabidopsis thaliana* miR159a control sequence (shControl) were mixed with pMDLg/prre, pRSVRev and pMD2.VSV-G packaging plasmids, and transfected into 70% confluent HEK293T cells using the calcium phosphate precipitation method. After 8 h, the media was changed, and viral supernatant was collected 24 h later and centrifuged at 20,000 rpm for 2 h at 4°C. The pellet was dissolved in 3% (v/v) lactose/PBS and stored at -70°C.

LNCaP and PC-3 cells were then transduced with concentrated lentiviral vectors that delivered shRNAs against ASCT2 or control sequence and expanded under puromycin selection (10 μg/mL) for at least 1 week. ASCT2 expression was examined by western blotting to confirm knockdown.

**Gene set enrichment analysis**

Gene set enrichment analysis (GSEA; http://www.broad.mit.edu/gsea) was used to identify gene clusters. Depending on sample size, phenotype or gene set permutation analysis with ratio-of-classes or signal-to-noise gene ranking was carried out. GSEA analyses generate a nominal P value, reflecting the statistical significance, estimated using an empirical phenotype-based permutation test. Venn diagrams were drawn using BioVenn (http://www.cmbi.ru.nl/cdd/biovenn/index.php). Published microarray data sets from Oncomine (v.4.4.3) were used for analysis of ASCT2 expression in patient cohorts.

**Supplementary Reference**:

1. Ettinger SL, Sobel R, Whitmore TG*, et al.* Dysregulation of sterol response element-binding proteins and downstream effectors in prostate cancer during progression to androgen independence. *Cancer Res* 2004; **64**: 2212-2221.

2. Qi J, Tripathi M, Mishra R*, et al.* The E3 ubiquitin ligase Siah2 contributes to castration-resistant prostate cancer by regulation of androgen receptor transcriptional activity. *Cancer Cell* 2013; **23**: 332-346.

3. Sadowski MC, Pouwer RH, Gunter JH*, et al.* The fatty acid synthase inhibitor triclosan: repurposing an anti-microbial agent for targeting prostate cancer. *Oncotarget* 2014; **5**: 9362-9381.

4. Wang Q, Beaumont KA, Otte NJ*, et al.* Targeting glutamine transport to suppress melanoma cell growth. *Int J Cancer* 2014; **135**: 1060-1071.
