# Supplementary figures and images for: Targeting ASCT2‐mediated glutamine uptake blocks prostate cancer growth and tumour development
Source: J Pathol. 2015 Apr 7;236(3):278–89. doi: 10.1002/path.4518 (PMC4973854; doi:10.1002/path.4518)

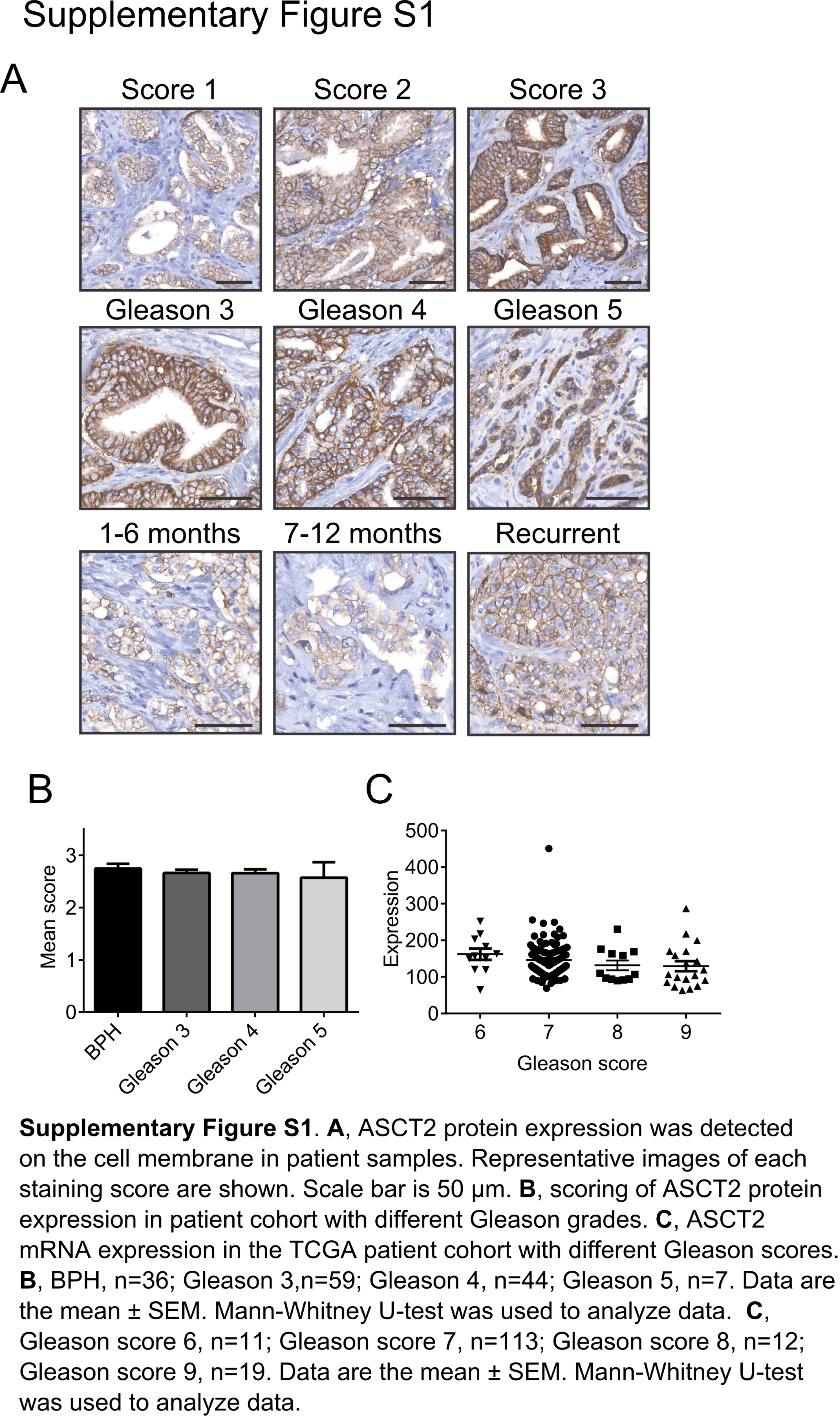

Supplement: Supplementary file 2 — FigureS1. A, ASCT2 protein expression was detected on the cell membrane in patient samples. Representative images of each staining score are shown. Scale bar is 50 urn. B, scoring of ASCT2 protein expression in patient cohort with different Gleason grades. C, ASCT2 mRNA expression in the TCGA patient cohort with different Gleason scores. B, BPH, n = 36; Gleason 3,n = 59; Gleason 4, n = 44; Gleason 5, n = 7. Data are the mean ± SEM. Mann‐Whitney U‐test was used to analyze data. C, Gleason score 6, n = 11; Gleason score 7, n = 113; Gleason score 8, n = 12; Gleason score 9, n = 19. Data are the mean ± SEM. Mann‐Whitney U‐test was used to analyze data. [file PATH-236-278-s002.tif]

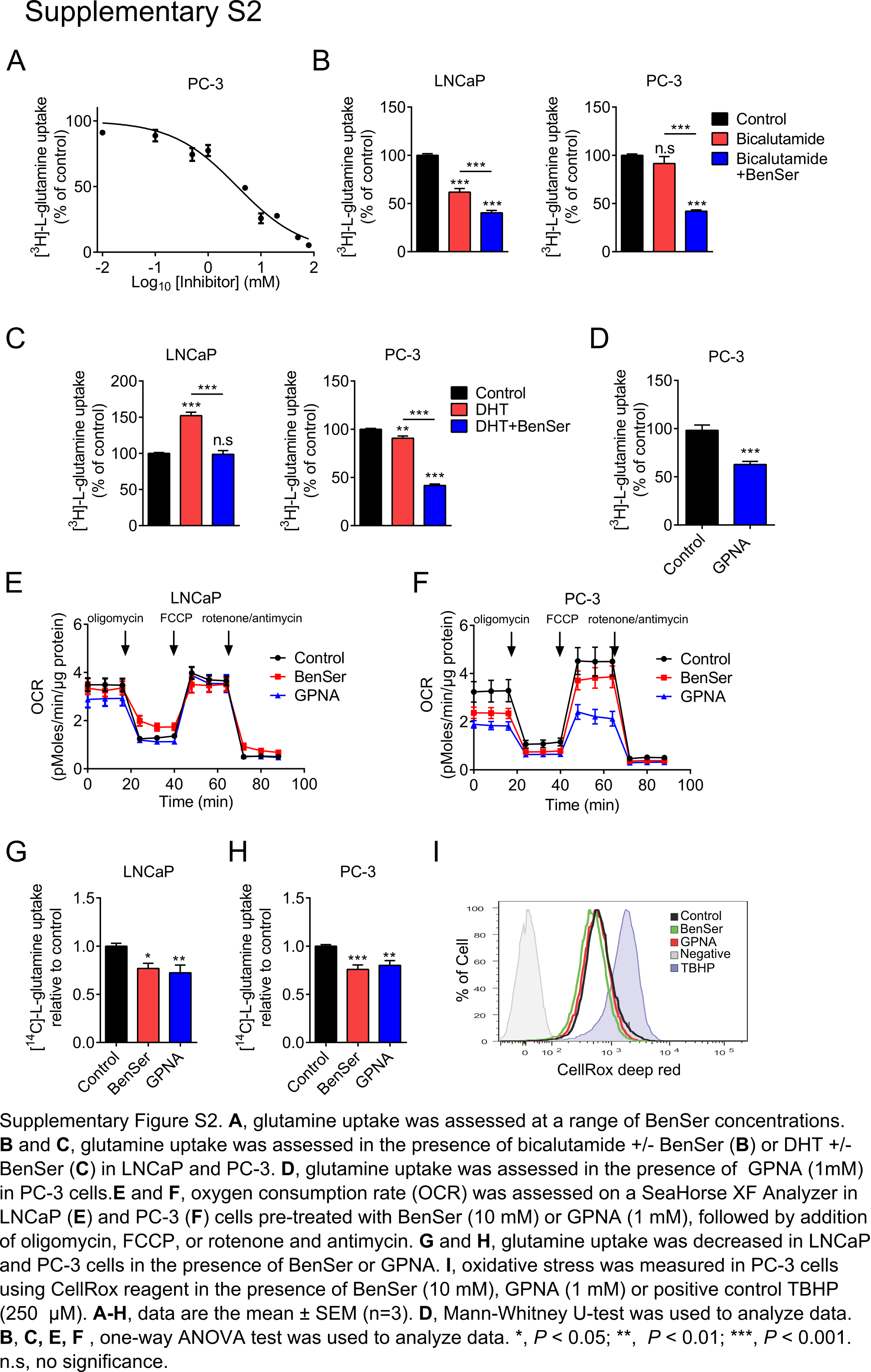

Supplement: Supplementary file 3 — A, glutamine uptake was assessed at a range of BenSer concentrations. Band C, glutamine uptake was assessed in the presence of bicalutamide +/‐ BenSer (B) or DHT +/BenSer (C) in LNCaP and PC‐3. 0, glutamine uptake was assessed in the presence of GPNA (1 mM) in PC‐3 cells.E and F, oxygen consumption rate (OCR) was assessed on a SeaHorse XF Analyzer in LNCaP (E) and PC‐3 (F) cells pre‐treated with BenSer (10 mM) or GPNA (1 mM), followed by addition of oligomycin, FCCP, or rotenone and antimycin. G and H, glutamine uptake was decreased in LNCaP and PC‐3 cells in the presence of BenSer or GPNA. I, oxidative stress was measured in PC‐3 cells using CellRox reagent in the presence of BenSer (10 mM), GPNA (1 mM) or positive control TBHP (250 ˜ M). A‐H, data are the mean ± SEM (n = 3). 0, Mann‐Whitney U‐test was used to analyze data. B, C, E, F , one‐way ANOVA test was used to analyze data. *, P < 0.05; **, P < 0.01; ***, P < 0.001. n.s, no significance. [file PATH-236-278-s003.tif]

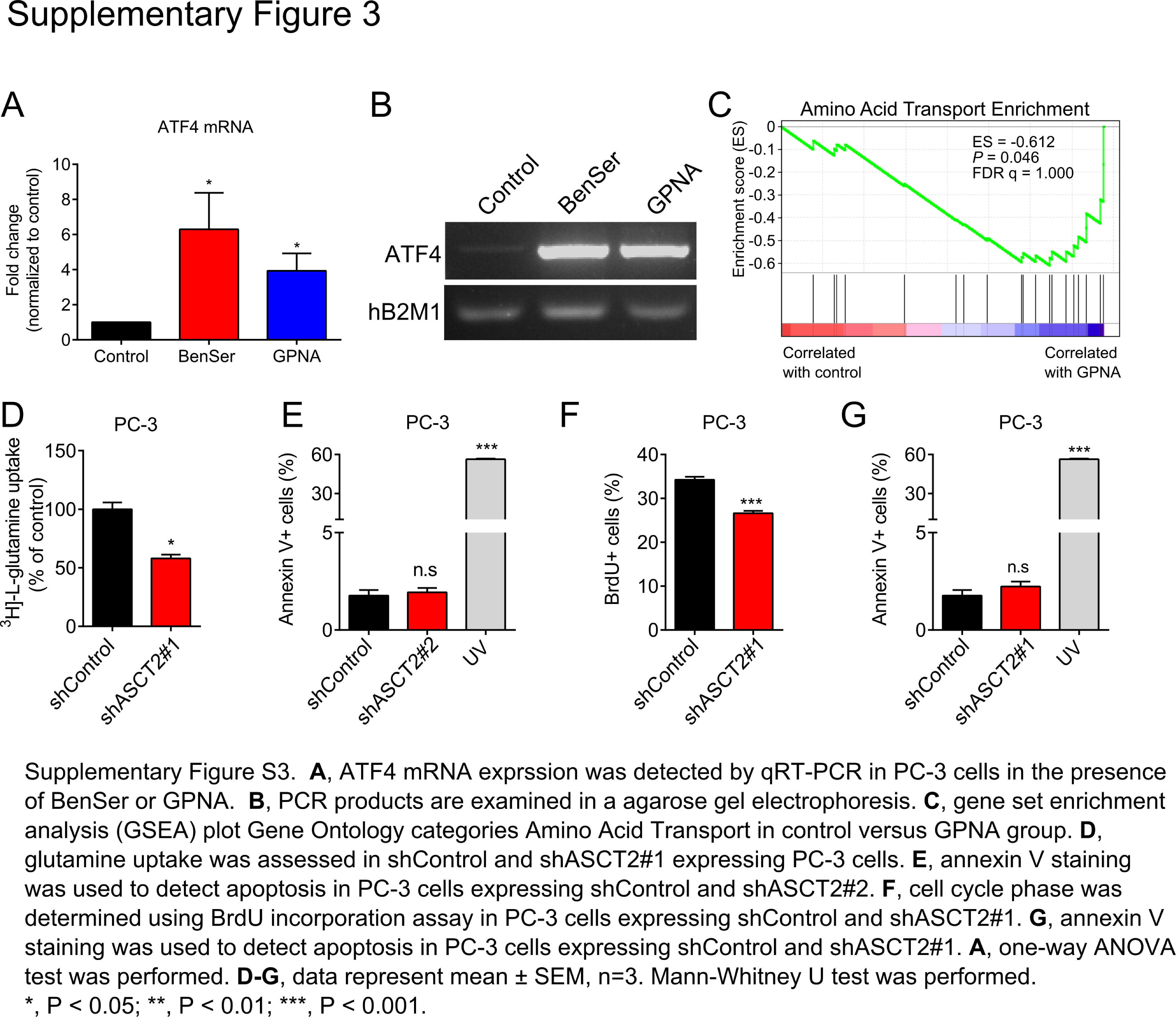

Supplement: Supplementary file 4 — A, ATF4 mRNA exprssion was detected by qRT‐PCR in PC‐3 cells in the presence of BenSer or GPNA. B, PCR products are examined in a agarose gel electrophoresis. C, gene set enrichment analysis (GSEA) plot Gene Ontology categories Amino Acid Transport in control versus GPNA group. 0, glutamine uptake was assessed in shControl and shASCT2#1 expressing PC‐3 cells. E, annexin V staining was used to detect apoptosis in PC‐3 cells expressing shControl and shASCT2#2. F, cell cycle phase was determined using BrdU incorporation assay in PC‐3 cells expressing shControl and shASCT2#1. G, annexin V staining was used to detect apoptosis in PC‐3 cells expressing shControl and shASCT2#1. A, one‐way ANOVA test was performed. O‐G, data represent mean ± SEM, n = 3. Mann‐Whitney U test was performed. *, P < 0.05; **, P < 0.01; ***, P < 0.001. [file PATH-236-278-s004.tif]

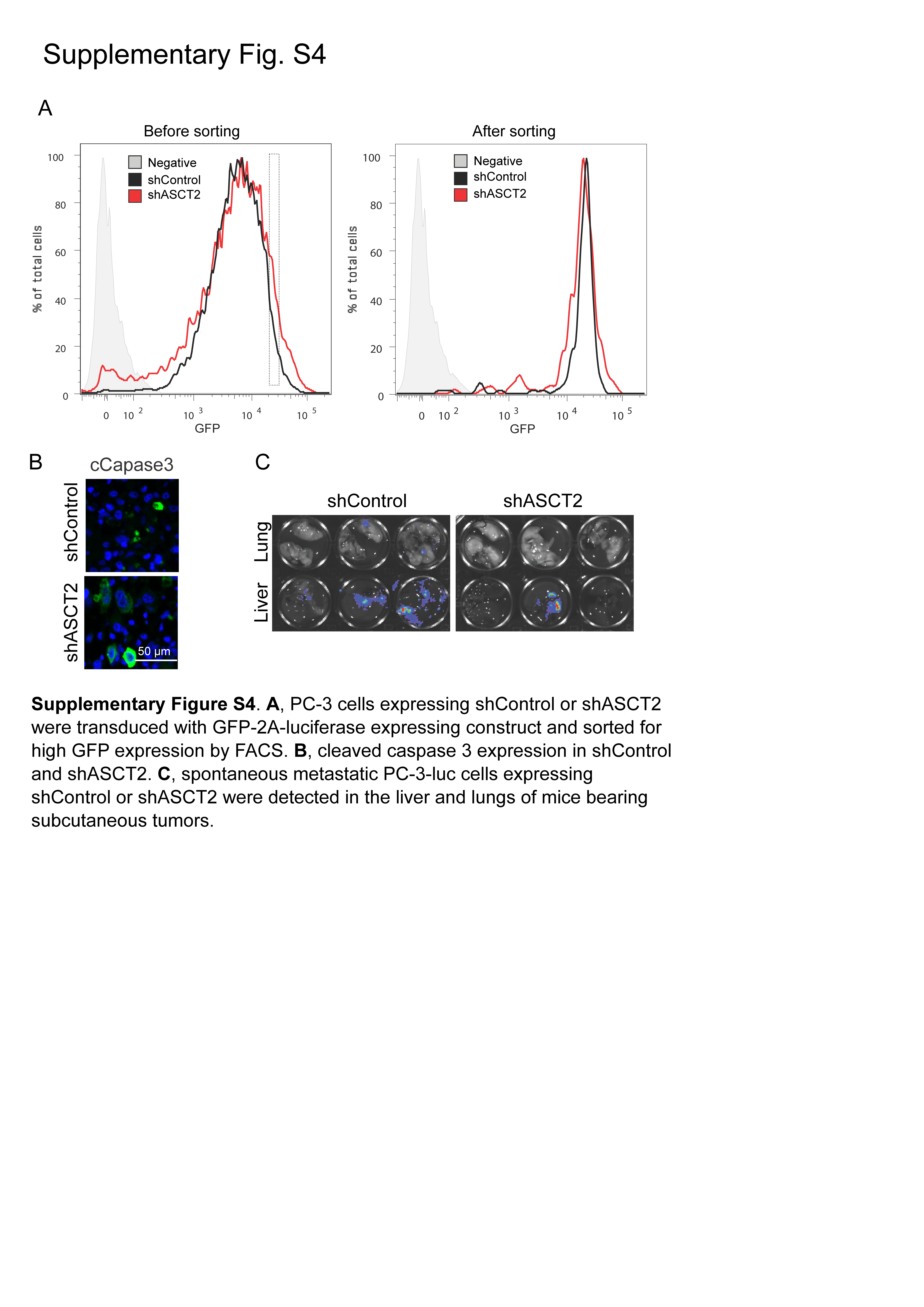

Supplement: Supplementary file 5 — FigureS4. A, PC‐3 cells expressing shControl or shASCT2 were transduced with GFP‐2A‐luciferase expressing construct and sorted for high GFP expression by FACS. B, cleaved caspase 3 expression in shControl and shASCT2. C, spontaneous metastatic PC‐3‐luc cells expressing shControl or shASCT2 were detected in the liver and lungs of mice bearing subcutaneous tumors. [file PATH-236-278-s005.tif]
